# Laser-based ultrasound interrogation of surface and sub-surface features in advanced manufacturing materials

**DOI:** 10.1038/s41598-022-07261-w

**Published:** 2022-02-28

**Authors:** Kathryn Jinae Harke, Nicholas Calta, Joseph Tringe, David Stobbe

**Affiliations:** Lawrence Livermore National Laboratory, Livermore, 94550 USA

**Keywords:** Engineering, Materials science, Physics

## Abstract

Structures formed by advanced manufacturing methods increasingly require nondestructive characterization to enable efficient fabrication and to ensure performance targets are met. This is especially important for aerospace, military, and high precision applications. Surface acoustic waves (SAW) generated by laser-based ultrasound can detect surface and sub-surface defects relevant for a broad range of advanced manufacturing processes, including laser powder bed fusion (LPBF). In particular, an all-optical SAW generation and detection configuration can effectively interrogate laser melt lines. Here we report on scattered acoustic energy from melt lines, voids, and surface features. Sub-surface voids are also characterized using X-ray Computed Tomography (CT). High resolution CT results are presented and compared with SAW measurements. Finite difference simulations inform experimental measurements and analysis.

## Introduction

Nondestructive characterization of defects in structures formed by advanced manufacturing (AM) methods is critical for aerospace, military, and high precision technologies where the presence of sub-surface defects can lead to degradation of structural integrity^1^. While certain defects such as surface cracks or pitting can be observed optically, sub-surface defects like voids or inclusions must be detected via nondestructive evaluation methods.

Laser powder bed fusion (LPBF) uses a laser to scan patterns over a bed of metal powder (typical particle diameter $\sim$ 15–100 $\upmu$m), heating the top powder layer to its melting temperature where it binds with layer below. A 3D object is formed when this process is repeated over many iterations^2–5^. While LPBF opens new possibilities for geometrically complex metal objects, one drawback is its propensity for forming sub-surface defects. These defects form from a variety of mechanisms, all of which are the result dynamic interaction of the laser with the liquid melt pool during the printing process^2–5^. Multi-physics modeling has been applied to understand these mechanisms^4,6^ and develop strategies to mitigate defect formation^2^. High speed, high resolution imaging approaches using both visible light^7,8^ and synchrotron X-rays^9–11^ have been applied to validate and complement multi-physics modeling approaches to understand defects in the melt pool^12^. However, no defect mitigation strategy can be completely successful, so approaches to characterize defects are still required. X-ray computed tomography (CT) is a useful method to measure these voids^13–16^, but long scan times and trade offs between part size and detection thresholds create challenges for applying CT as a universal inspection tool in a production environment. To complement post-build CT inspection for defect quantification, many studies have applied different in situ process monitoring approaches to detect defects as they form, including techniques that interrogate the entire build at once^17,18^ or only the melt pool itself^19–22^. For more details about in situ process monitoring for AM, the reader is directed to reviews on the topic^23^.

Here we propose an all-optical ultrasound system suitable for in situ characterization of single LPBF melt lines. Laser based ultrasound (LBU) has previously been studied for use on AM samples including for detecting residual stresses^24^, subsurface defects using bulk waves^25^, surface defects using resolved acoustic spectroscopy^26^, and subsurface features using Rayleigh and Lamb waves^27^. Rayleigh (surface acoustic) waves are promising for characterizing laser melt lines because they are sensitive to surface and near surface features. Surface acoustic waves (SAW) have been used to characterize surface and near-surface features including cracks^28–32^, pits^33,34^, welds^35,36^, and steps and notches^37–39^. SAW are also used in seismology, at a much larger length scale, for detecting near-surface structures such as caves^40–45^. SAW are readily excited, via the thermoelastic effect, with pulsed laser light and can be detected remotely using an interferometer^46–49^. This allows the implementation of an all-optical diagnostic which can be realized on a LPBF platform for on-demand characterization. In contrast to other more conventional nondestructive evaluation techniques like X-ray CT, LBU is better posed to perform real-time inspection and can acquire and process data at a faster rate than X-ray CT. With the use of a kHz repetition rate laser, LBU scans can be performed on the time scale of single minutes as compared to several hours or days for a X-ray CT scan with sufficient resolution to visualize the same defect size of interest^50^.

Next, we present certain physical properties of surface waves to demonstrate the strengths and limitations of SAW for sub-surface measurements. For simplicity, we assume surface waves on an isotropic, homogeneous, linear elastic half-space. Surfaces waves are nondispersive, meaning that the phase and group velocities are equivalent and equal to the Rayleigh speed, $c_{R}$, which is well-approximated by^51^:

1 $$\begin{aligned} c_{R} \approx c_{T}\frac{0.862+1.14\nu }{1+\nu } \end{aligned}$$

where $\hbox {c}_{{T}}$ is the shear wave velocity and $\nu$ is Poisson’s ratio. As such, a broadband pulse of acoustic energy can propagate without shape distortion, allowing distal interrogation and interpretation of scattered energy without the added complexity of frequency dispersion. Another property of surface waves is that the displacement and stress decay exponentially with depth^51^, limiting the depth sensitivity to approximately one wavelength ($\lambda _{R}$) below the surface^52^. Here we explore the depth sensitivity quantitatively by considering the time-averaged power flux density ($\left\langle S\right\rangle$) in the direction of wave propagation (x) as a function of depth (z) and frequency ($\omega$):

The average power flux density^53^ is described as $\left\langle S\right\rangle$, where the complex conjugation is denoted as, $*$.

2 $$\begin{aligned} \left\langle S \right\rangle =-\frac{1}{2}Re \left[ i\omega \left\{ T_{11}x^{*}+T_{31}z^{*} \right\} \right] \end{aligned}$$

Which is composed of the nonvanishing components of the stress dyad as $T_{11}$ and $T_{31}$.

3 $$\begin{aligned} T_{11}= & {} (\lambda +2\mu )\frac{\partial ^2 \phi }{\partial x^2}+\lambda \frac{\partial ^2 \phi }{\partial z^2}-2\mu \frac{\partial ^2 \psi }{\partial x\partial z} \end{aligned}$$

4 $$\begin{aligned} T_{31}= & {} \mu \left[ 2\frac{\partial ^2 \phi }{\partial x\partial z} + \left( \frac{\partial ^2 }{\partial x^2} - \frac{\partial ^2 }{\partial z^2}\right) \psi _{2} \right] \end{aligned}$$

where $\lambda$ and $\mu$ are the Lamé’s constants and the displacements, $u_{x}$ and $u_{z}$ can be written in terms of the two potentials, $\phi (x,z)$ and $\psi _{2}(x,z)$.

5 $$\begin{aligned} u_{x}= & {} \frac{\partial \phi }{\partial x}-\frac{\partial \psi _{2}}{\partial z} \end{aligned}$$

6 $$\begin{aligned} u_{z}= & {} \frac{\partial \phi }{\partial z}+\frac{\partial \psi _{2}}{\partial x} \end{aligned}$$

7 $$\begin{aligned} \phi= & {} \frac{A}{2i k_{R} \alpha _{L}}e^{\alpha _{L}z}e^{i k_{R} x} \end{aligned}$$

8 $$\begin{aligned} \psi _{2}= & {} \frac{A}{(\alpha _{T}^{2}+ k_{R} ^{2})}e^{\alpha _{T}z}e^{i k_{R} x} \end{aligned}$$

where $\alpha _{L}=(k_{R} ^{2}-\omega ^{2}/c_{L}^{2}))^{1/2}$, $\alpha _{T}=(k_{R}^{2}-\omega ^{2}/c_{T}^{2}))^{1/2}$, $k_{R}$ is the wavenumber, and $c_{L}$ is the longitudinal wave velocity.

**Figure 1:**
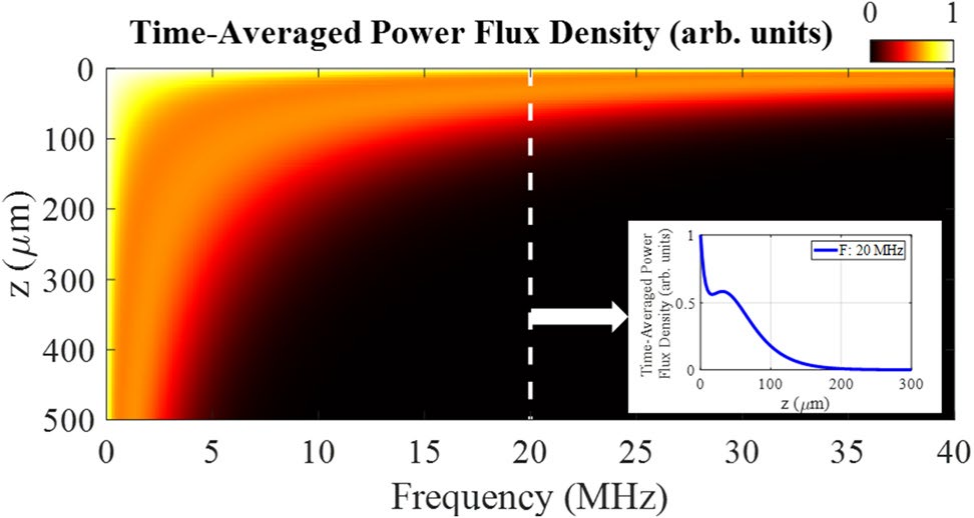
Time-averaged power flux in the direction of wave propagation (x) as a function of frequency and depth into surface (z), using $\lambda$ = 129 GPa, $\mu$ = 41 GPa, and $\rho$ = 4.42 g/cc. The inset shows the time-averaged power flux at 20 MHz.

Figure 1 shows the clear dependence of power flux on depth and frequency. In general, as frequency increases the power flux is more confined to the surface. The inset in Fig. 1 shows the time-averaged power flux density versus depth at 20 MHz ($\lambda _{R}$ = 142 $\upmu$m). Nearly 50% of the power flux is lost in the first 15 $\upmu$m. The power flux increases slightly around 30 $\upmu$m, corresponding to a depth where the in-plane displacement changes signs and the elliptical orbits reverse direction^54^. After this local maximum, the power flux decays exponentially and is reduced to about 5% of the peak flux at a depth equal to $\lambda _{R}$.

For sub-surface feature detection it is also necessary to consider the size of the defect. In general, the minimum detectable defect size depends on the acoustic wavelength, where smaller defects will be more sensitive to shorter wavelengths (higher frequencies). Higher frequencies, however, are less penetrating into the sample. In this work, we demonstrate the implementation of an all-optical SAW system for characterizing single track laser melt lines in a Titanium alloy (Ti-6Al-4V). Surface features, including the laser melt line, breaks in the melt line, and metal spatter, are independently measured by optical imaging. Sub-surface voids are independently measured using X-ray computed tomography (CT). Cracks were not detected and are not considered in this work.

## Methods

### Ti-6Al-4V sample single track laser melting

**Figure 2:**
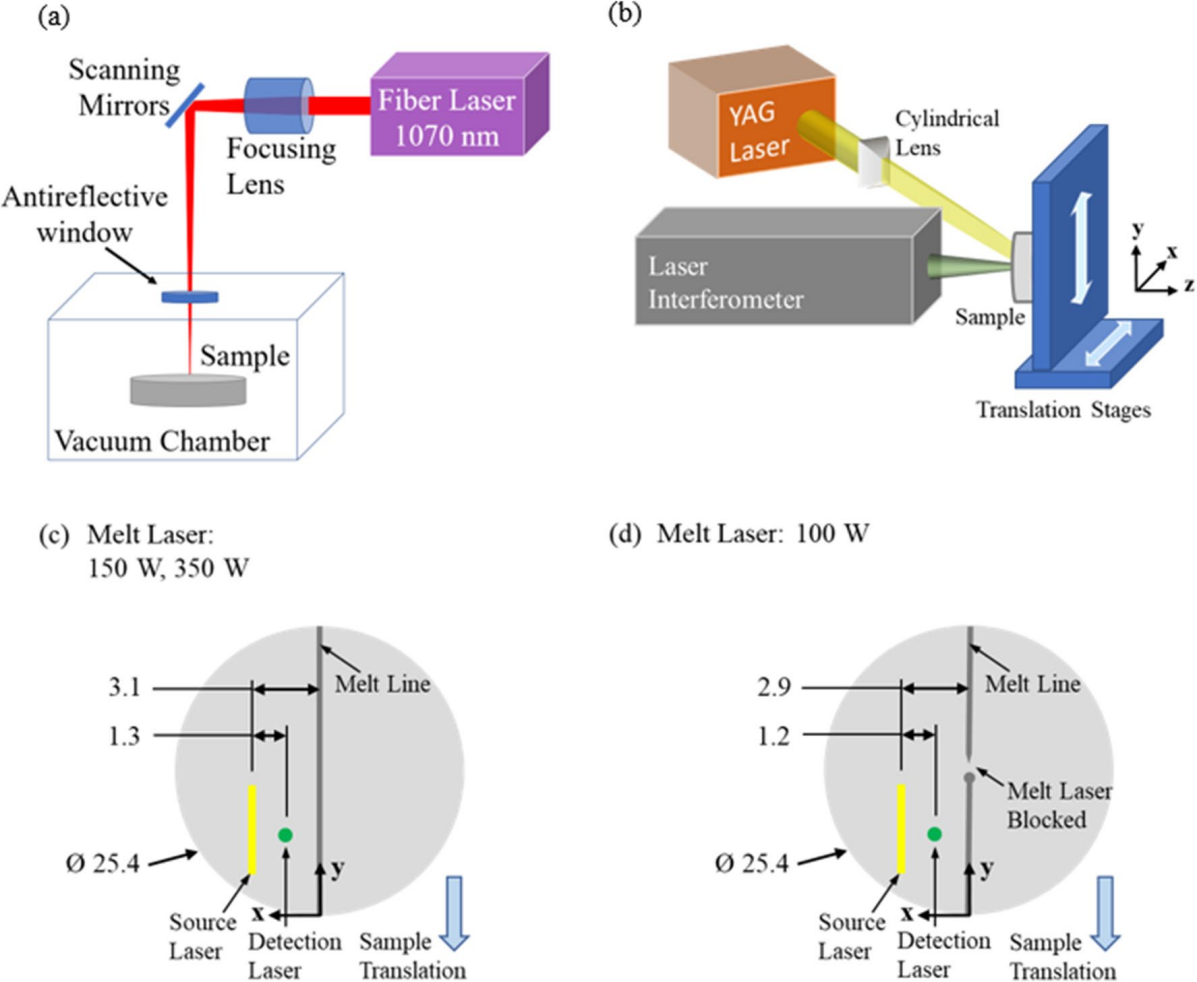
(**a**) Experimental setup for producing the laser melted line scans. (**b**) Experimental setup for generating and detecting surface acoustic waves. A pulsed laser is used to generate ultrasound and the displacement is measured using a photorefractive interferometer. (**c**,**d**) Geometry of sample(s) and location of source and detection relative to the melt line (units in mm). The melt line is scanned by translating the sample in the -y direction.

The experimental configuration for producing the laser melted lines in a Ti-6Al-4V sample is shown in Fig. 2a. A 600 W fiber laser (JK lasers, model JK600FL) was directed through a 3-axis galvanometer scanner (Nutfield technologies) and into a $15 \times 15 \times 15$ $\hbox {cm}^{3}$ vacuum chamber through a high purity fused silica window. The 600 W build laser is focused to a circular Gaussian shape with a diameter of 50 $\upmu$m. This diameter uses the D4$\sigma$ definition, where a 50 $\upmu$m diameter circle contains 4 times the standard deviation of the intensity distribution, or 95% of the laser intensity for a Gaussian beam. The chamber was evacuated and then back-filled with Ar gas as an inert process environment. Melting experiments were performed under a $\sim$760 Torr Ar atmosphere. Tracks were melted on the polished top surface of a 25.4 mm diameter Ti-6Al-4V cylinder that was roughly 12 mm in height. Each sample contained a single melt track that extended across the surface of the entire sample. Melting experiments were performed at laser powers of 100, 150, and 350 W and scan speeds of 500 mm/s. These conditions were chosen to ensure the melt pool was in an unstable keyhole condition with a variety of melt pool depths. This leads to pore formation caused by melt pool instabilities at random locations along the melt track length. The depth of these pores correlates with the overall melt pool depth. For the 100 W sample, a razor blade was placed on top of the substrate surface to simulate a scenario in which the laser was briefly obscured by spatter or another obstruction during the scan.

### Laser-based ultrasound

**Figure 3:**
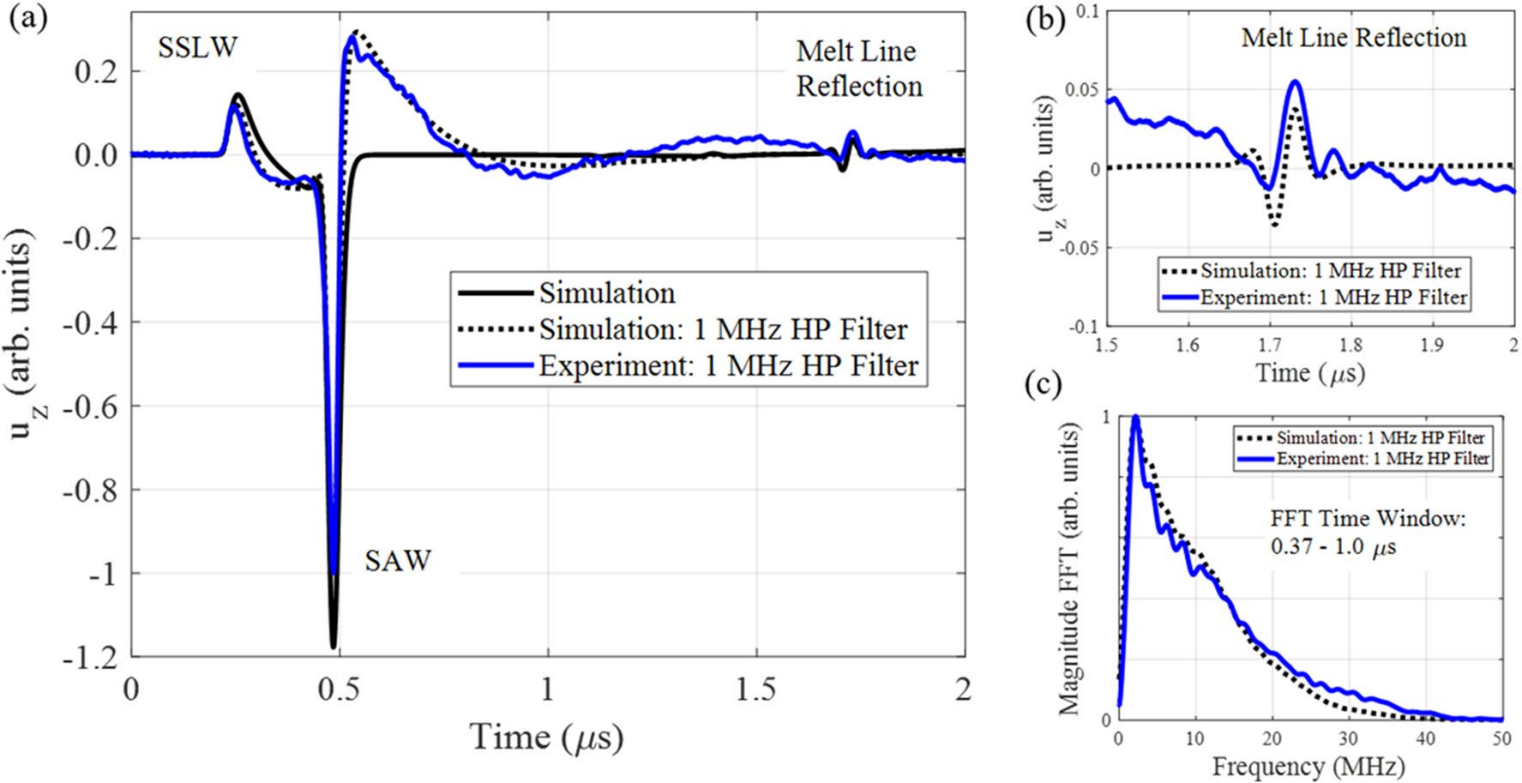
(**a**) Simulated and experimentally measured normal displacement from the pulsed laser excitation. (**b**) Zoomed-in portion of waveform in (**a**) showing the measured scattering from the melt line. (**c**) Magnitude of the Fourier transform of the surface acoustic wave time windowed between 0.37 and 1.0 $\upmu$s.

Figure 2b shows a schematic of the experimental design for generating and detecting surface acoustic waves (SAW). SAW are generated with a thermoelastic source from a Q-switched Nd:YAG laser which provides 1.5 mJ, 15 ns pulses. The unfocused laser spot was Gaussian with a full width at half max (FWHM) of 7.4 mm. A cylindrical lens focused the light to a line with a FWHM of 70 $\upmu$m measured along the x-direction at the sample surface. The normal displacement was detected using a commercial multi-channel random quadrature photorefractive interferometer (Sound&Bright, Quartet, 532 nm) with a calibrated linear response (1 nm / 100 mV) over the detection bandwidth (1–100 MHz). The output of the interferometer was digitized and recorded on an oscilloscope with a sampling frequency of 1 GHz. Samples were mounted on two orthogonal linear translation stages. Figure 2b,c shows the sample geometry, location of the source and detection, and features present. Each sample has a laser-generated melt line which spanned the entire sample diameter starting at the axis origin as shown in Fig. 2c,d. Initially, the generation laser spot and detection laser spot are located at approximately 3.0 mm and 1.8 mm, respectively, from the melt line in the x-direction and at y = 4 mm. During each experiment, the sample is translated 18 mm in the -y direction in 25 $\upmu$m steps, collecting SAW measurements at each step, resulting in a scan from y = 4 mm to y = 22 mm. At each step, 500 waveforms are averaged and then the signal is recorded. Figure 3a shows a single waveform, collected at y = 13 mm, on the 150 W melt line sample together with simulation results. Simulations were performed using a commercial finite element time domain software package (On-Scale, PZ-Flex), to inform the experimental measurements and assist with interpreting the experimental results. The simulation used a $c_{L}$ of 6.204 mm/$\upmu$s, a $c_{T}$ of 3.044 mm/$\upmu$s, and a density ($\rho$) of 4420 kg/m$^{3}$. The sample was modeled on an orthogonal grid with element dimensions $0.1 \times 0.1$ $\upmu$m$^{2}$ (x, z) and plane strain in the y-direction.

The melt line protrusion above the surface was modeled as a circular segment with a chord length and height of 100 $\upmu$m and 20 $\upmu$m, respectively. These dimensions were calculated by fitting a circular segment to structured light measurements of the actual 150 W sample melt line. The forcing function was derived from a thermoelastic Gaussian laser source with a 70 $\upmu$m FWHM^55^. The center of the source is located on the top of the sample at x = 3.0 mm and the displacement in the z-direction is measured at x = 1.7 mm, relative the coordinate system shown in Fig. 2c,d. Figure 3a shows the measured displacement over 2 $\upmu$s, where the surface-skimming longitudinal wave (SSLW), SAW, and reflection from the melt line are labeled. For the simulation result, both the raw data and the data after processing with a 1 MHz high pass (HP) filter are shown. The filtered data is shown to facilitate comparison with the experimental measurement which has a lower frequency detection limit of 1 MHz. For this reason, all presented simulation results for the remainder of this work have been processed with a 1 MHz HP filter. Figure 3b shows the measured reflection from the melt line is shown in detail. The time delay between the incident SAW and reflected signal (1.2 $\upmu$s) is consistent with twice the distance between the detection and the center of the melt line (2$\cdot$1.7 mm) divided by the SAW velocity ($c_{R}$) 2.842 mm/$\upmu$s. The measured reflection from the melt line is a SAW reflected from the incident SAW. Figure 3c shows the magnitude of the fast Fourier transform (FFT) of the incident SAW after time windowing the signals in Fig. 3a from t = 0.37–1.0 $\upmu$s with a Tukey (tapered cosine) window with a cosine fraction (*r*) of 0.2. The incident SAW is broadband in frequency. Figure 3a–c demonstrate excellent agreement between simulation and experimental measurement of the laser-based SAW system.

**Figure 4:**
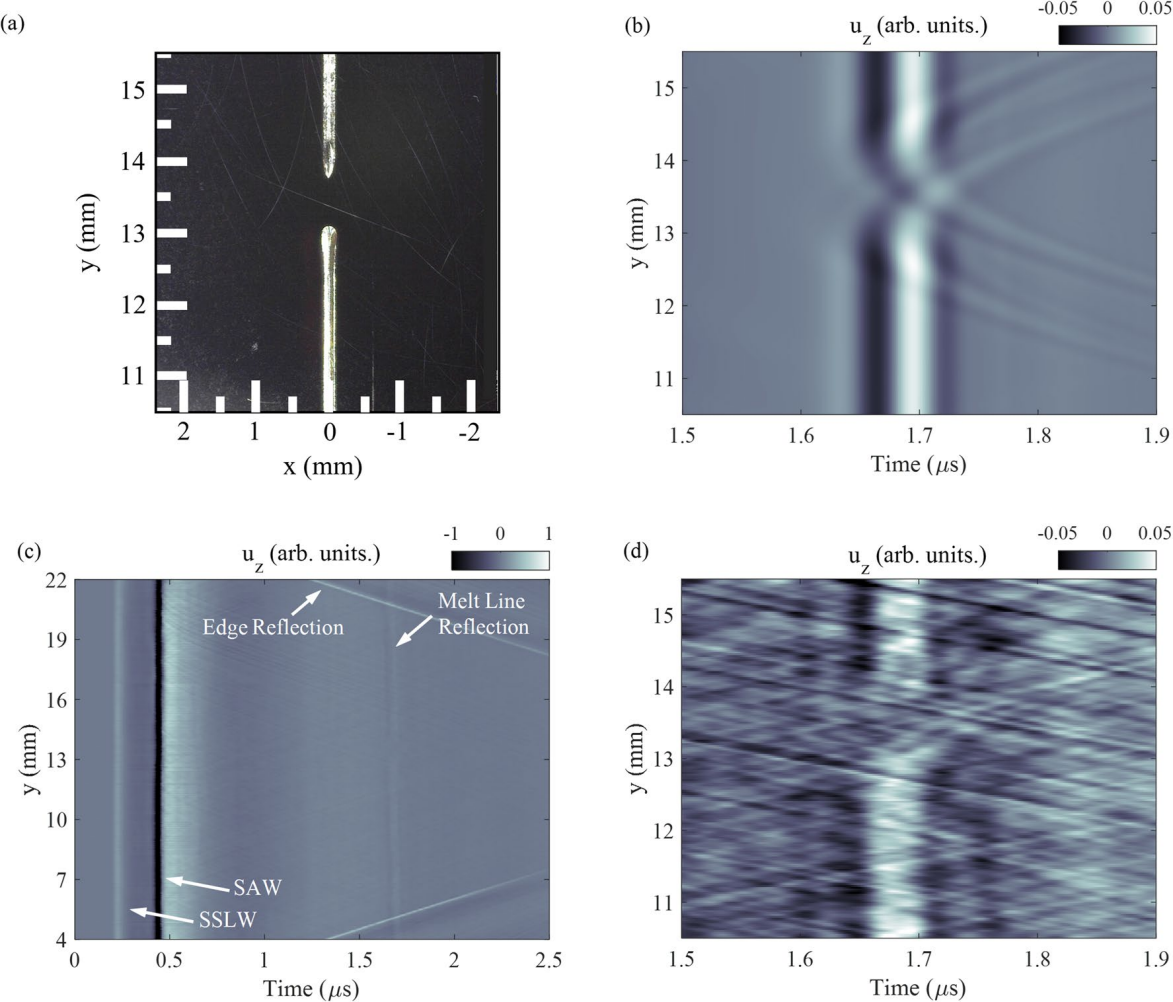
(**a**) Optical image of melt line. A break in the melt line is observed near y = 13 mm due to interruption of the beam by the edge of a razor-blade. (**b**) Simulation of displacement near break in the melt line. Parabolic scattering from the end and start of the melt line are observed as well as a gap in the planar reflection from the melt line. (**c**) Experimentally measured normal displacement while scanning along the y-axis of the sample. A break in the planar reflection from the melt line is apparent near y = 13 mm. (**d**) Magnified portion of the image in (**c**) showing parabolic scattering from the end of the melt line,faint parabolic scattering from the start of the melt line and the gap in the melt line.

Three Ti-6Al-4V samples were created with different melt laser powers. First, a Ti-6Al-4V sample was created with a melt laser power of 100 W with a small break in the melt line to assess the laser-based SAW system sensitivity to defects in the melt line. The sample was created using the previously-described configuration; however, a razor blade was placed such that the thin edge of the blade obstructed the melt laser. An optical image of the sample near this location is shown in Fig. 4a where an approximately 0.75 mm break in the melt line is clearly observed near y = 13 mm. At the start of the break, the melt line terminates in a half sphere, whereas at the end of the break the melt line starts with an arrow shape. The sample was measured using the previously-detailed laser-based SAW setup. The part was scanned from y = 4–22 mm in 25 $\upmu$m steps.

A second Ti-6Al-4V sample was created with a melt laser power of 150 W with a single continuous melt line, as shown in Fig. 1c, and the configuration previously described. The surface of the sample was measured using structured light^55^. After measurement by the laser-based SAW setup, the sample was sectioned at x = ± 1.5 mm and z = 1.5 mm, resulting in a $3.0 \times 25.4 \times 1.5$ $\hbox {mm}^{3}$ (x, y, z) sample for X-ray CT.

A third Ti-6Al-4V sample was created with a melt laser power of 350 W with a single continuous melt line, as shown in Fig. 1c. First the sample melt line topology was measured with structured light; then the sample was inspected using a confocal microscope. Finally, the sample was characterized with SAW. A section of the 350 W sample, containing the melt line and similar in size and location as detailed above, was removed for independent measurement by X-ray CT.

### X-ray computed tomography sub-surface void characterization

The Zeiss Xradia 510 Versa X-ray CT system was used for the characterization of sub-surface void location and size in the 150 W and 350 W Ti-6Al-4V samples. Due to the limited field of view ($\sim$ 3.9 mm $\times$ 3.9 mm) at the desired 4 $\times$ magnification, each sample (3.0 $\times$ 25.4 $\times$ 1.5 $\hbox {mm}^{3}$ (x, y, z)) was scanned and then translated vertically in $\sim$3 mm increments to capture the full region of interest along the melt line (y = 6–21 mm). The 150 W and the 350 W samples were each scanned in five different sections. For the X-ray CT scans, each sample was mounted vertically with the axis of rotation oriented along the sample’s y-axis and rotated 180$^{\circ }$ + 1.80$^{\circ }$ (fan beam angle) through 1601 projections. A 4X magnification objective was used for all CT scans with voltage = 120 kV, power = 10 W, exposure time = 22 s, binning = 1, source filter = HE2, source-to-object distance = 62 mm, detector-to-object distance = 48 mm, multi-reference. The total scan time was 22h:49m:20s per section.

Scout-and-$\hbox {Scan}^{TM}$ Control System Reconstructor 14.0.14829 was used to reconstruct the samples and TXM3DViewer was used to visualize and analyze the reconstructed datasets. The reconstruction of the Ti-6Al-4V samples was optimized with respect to the center shift and the beam hardening constant. A general smoothing factor of 1 was applied to all reconstructions. The approximate voxel size in the reconstruction was 1.92 $\upmu \hbox {m}^{3}$/voxel. The void analysis was performed in TXM3DViewer to determine the void distance along melt line (y (mm)), void depth (z ($\upmu$m)), and void diameter (d ($\upmu$m)) for both the 150 and 350 W samples.

## Results

### Laser-based ultrasound

The 100 W Ti-6Al-4V sample with a break in the melt line was modeled using the previously-detailed material parameters and structured light measurements of the melt line and break. The cross section of the melt line was measured to be, on average, a circular segment with chord length and height 100 $\upmu$m and 15 $\upmu$m respectively. The break in the melt line was between y = 13.0 and y = 13.75 mm. The circular and arrow-shaped geometry at the stop and start of the melt line, respectively, were also characterized using structured light. The sample was modeled on a 5 $\times$ 5 $\times$ 5 $\upmu \,\hbox {m}^{3}$ (x, y, z) grid where the laser-based SAW excitation and detection system was simulated. Figure 4b shows the scattered SAW from the melt line, near the break. Here we observe a gap in the planar reflection corresponding to the break in the melt line, as well as parabolic scattering from the stop and start of the melt line. The scattering from the end of the melt line is more pronounced due to the different geometries at the stop and start of the melt line.

Figure 4c shows the experimentally-measured surface normal displacement ($u_{z}$) where the incident SSLW and SAW are labeled as well as the edge and melt line reflections. The incident SAW scattering from the melt line is measured as a planar reflection near 1.7 $\upmu$s with the exception of a short gap around y = 13 mm. Figure 4d shows the SAW reflection around y = 13 mm. A gap in the melt line reflection is apparent near y = 13 mm along with parabolic scattering from the end of the melt line. There also appears to be parabolic scattering from the start of the melt line, at a lower magnitude than at the stop. Experimental measurements show good agreement with the simulation results and with the optical measurements of the melt line geometry.

**Figure 5:**
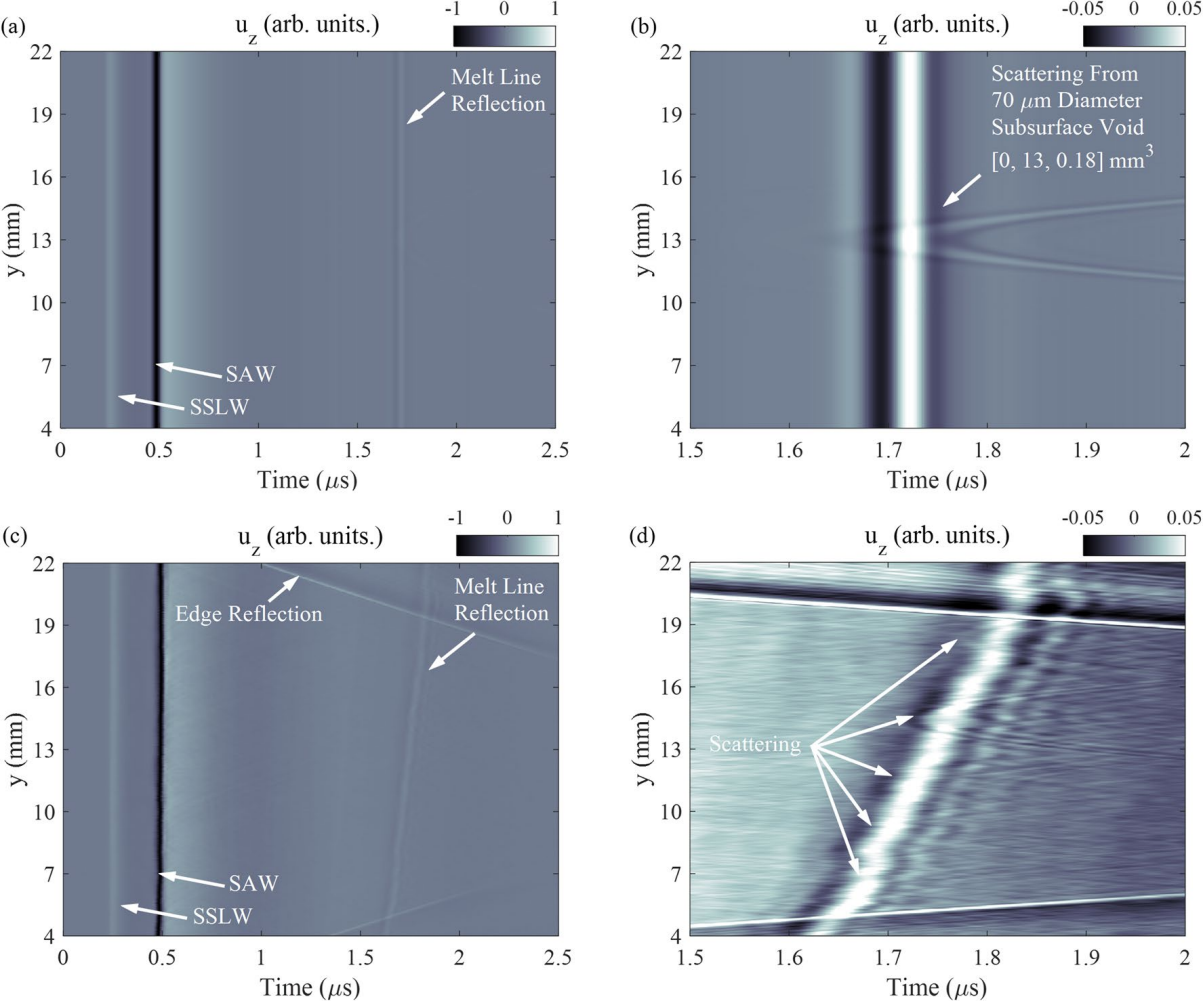
(**a**) Simulation of normal displacement while scanning along the y-axis of the sample. (**b**) Zoomed-in portion of the image in (**a**) showing planar scattering from the melt line and parabolic scattering from a 70 $\upmu$m diameter sub-surface void located at (0, 13, 0.18) $\hbox {mm}^{3}$ (x, y, z). (**c**) Experimentally measured displacement while scanning along the y-axis of the sample. (**d**) Zoomed-in portion of the image in (**c**) showing planar scattering from the melt line and scattering from multiple sub-surface voids.

The 150 W Ti-6Al-4V sample was modeled using the previously-detailed material parameters as well as structured light measurements of the melt line. The cross section of the melt line was measured to be, on average, a circular segment with chord length and height 100 $\upmu$m and 20 $\upmu$m respectively. We also simulated a single sub-surface spherical void, 70 $\upmu$m diameter, located at (0, 13, 0.18) mm (x, y, z). Figure 5a shows the surface normal displacement with SSL, SAW, and melt line reflections labeled. The incident SAW scattering from the melt line is observed as a planar reflection near 1.7 $\upmu$s. Figure 5a shows a magnified portion of the displacement field in Fig. 5b with observable scattering from the subsurface void. Scattering from the void is parabolic; the location of the parabola’s vertex along the scan axis (y-axis) is coincident with the void location (y = 13 mm).

Figure 5c shows the experimentally-measured surface normal displacement is shown with the SSL, SAW, and melt line and edge reflections labeled. The reflection from the melt line is near 1.7 $\upmu$s, planar with a slight tilt with respect to the y-axis due to the alignment of the sample relative to the translation stage. Reflections from the outer edge of the sample are also present near the top and bottom of the scan. Figure 5d shows the measured displacement near the melt line reflection. The reflection from the melt line is consistent with the simulation except for the previously-noted alignment deviation. Furthermore, several distinct parabolic scattering locations are observed, each of which has its vertex located on the melt line reflection. With respect the y-axis, the vertices of the most distinct parabolic scattering are at y = 14.2 and 18.4 mm. There are also some less distinct parabolic scattering locations with vertices at y = 7.0, 9.4, and 11.7 mm. Finally, incoherent scattering is observable just after the melt line reflection along the entire scan axis.

**Figure 6:**
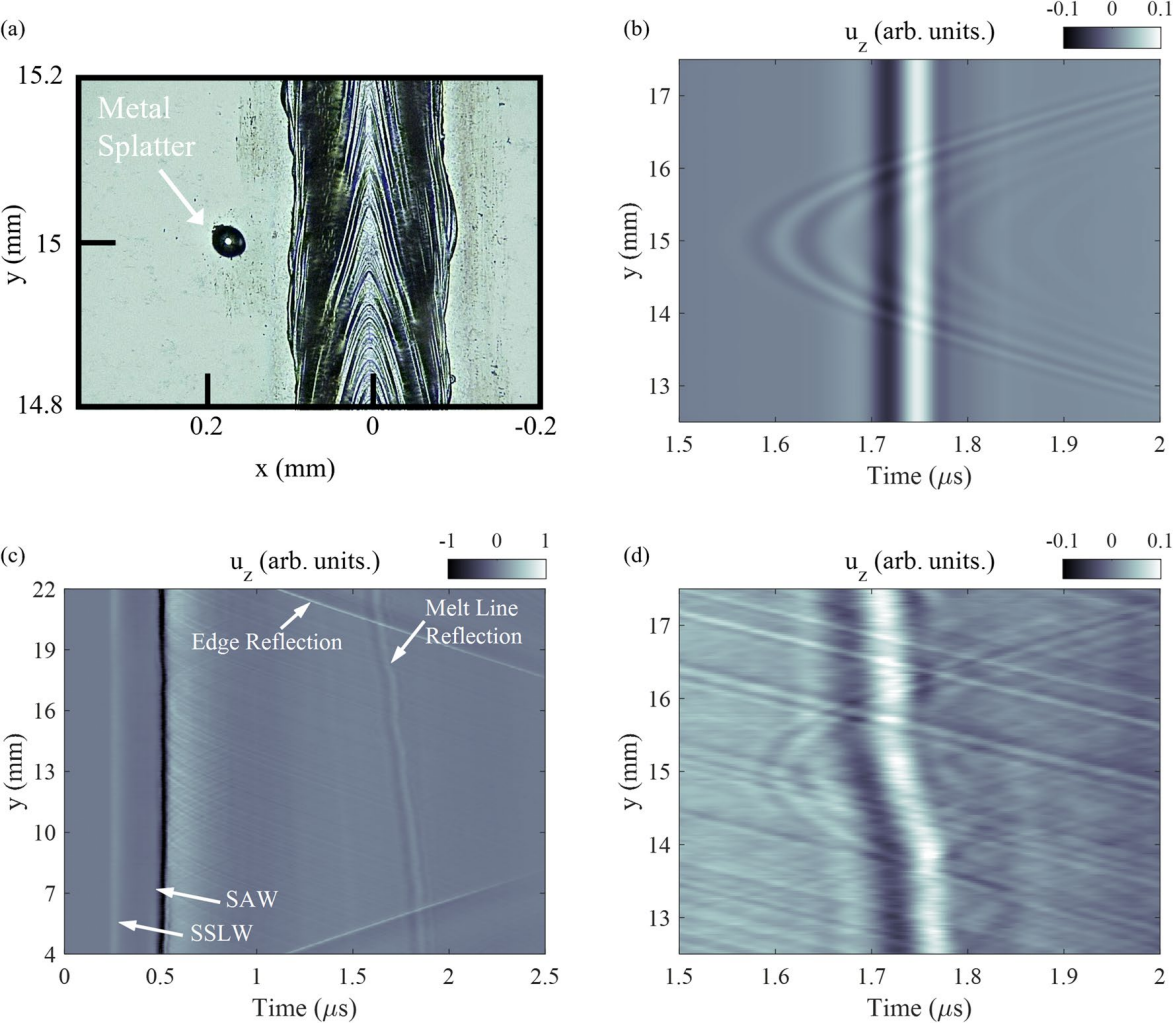
(**a**) Confocal microscope image of metal spatter that occurred during the laser melting processing. The metal spatter is located approximately 200 $\upmu$m from the melt line and is indicated with an arrow. (**b**) Simulation of displacement near metal spatter. Parabolic scattering is observed prior to the melt line reflection. (**c**) Experimentally measured normal displacement while scanning along the y-axis of the sample. (**d**) Zoomed-in portion of the image in (**c**) showing parabolic scattering from metal spatter prior to the melt line reflection.

The 350 W Ti-6Al-4V sample was modeled using the previously detailed-material parameters as well as structured light measurements of the melt line. The melt line shape was measured to be, on average, a circular segment with a chord length and height of 150 $\upmu$m and 35 $\upmu$m, respectively. Several large metal spatter deposits were also identified adjacent to the melt line. One of these deposits was measured with a confocal microscope as seen in Fig. 6a. This metal deposit was near y = 15 mm and x = 0.2 mm, a half sphere of approximately 50 $\upmu$m diameter. This geometry was used to model the sample.

Figure 6b shows the simulated displacement field near the metal deposit where parabolic scattering is observed from the metal deposit in addition to the planar reflection from the melt line. The location of the deposit relative to the melt line can be calculated using the difference in the time of arrival between the vertex of the parabolic scattering and the melt line reflection and the $\hbox {c}_{{R}}$.

**Figure 7:**
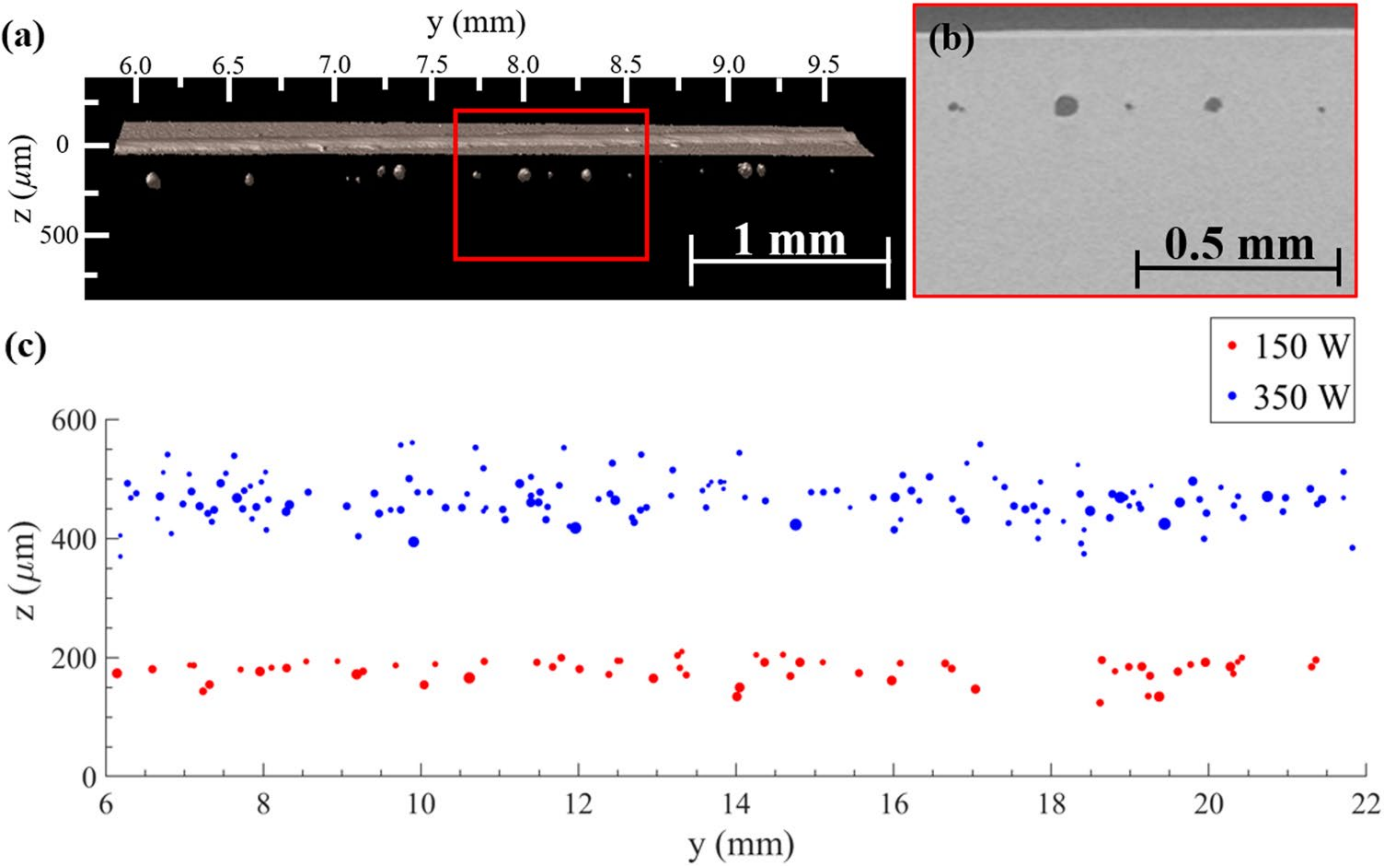
(**a**) A view of the X-ray CT reconstruction of the 150 W sample in the range y = 6.00–9.78 mm. The red box outlines the sectioned view shown in (b). (**b**) A 2D slice through the YZ plane of the X-ray CT reconstruction. (**c**) The sub-surface void locations for the 150 W and 350 W Ti-6Al-4V samples found with X-ray CT. Described by the void location along the track (y (mm)), void depth below the track line (z ($\upmu$m)), and void diameter (d ($\upmu$m)).

Figure 6c shows the experimentally-measured displacement with labeled incident SAW, SSLW, and reflection from the edges and melt line. There is a slight angle in the planar reflection from the melt line due to sample alignment relative to the translation axis. Figure 6d shows the measured displacement field near the deposit location. Here parabolic scattering is observed with the vertex aligned with the location of the deposit along the y-axis. The experimental and simulation scattering measurements from the half sphere metal deposit are in excellent agreement. The X-ray CT measurements revealed the presence of multiple subsurface voids as depicted in Fig. 7. These voids were on average 25 $\upmu$m in diameter and were located on average 466 $\upmu$m below the sample surface. There was no observed coherent scattering from any subsurface voids in the SAW experimental data. This finding is consistent with the power-flux density calculation in Fig. 1 where very little power penetrates z > 400 $\upmu$m for frequencies above 5 MHz ($\lambda _{R}$ $\sim$ 568 $\upmu$m). This result was also confirmed via simulation where a 25 $\upmu$m diameter void located at z = 400 $\upmu$m could not be detected above the experimental noise level. The experimental data does show some features in the immediate wake of melt line reflection. However, these features are not consistent with distinct parabolic scattering from individual voids and may be due to surface features of the melt line itself.

### X-ray computed tomography

X-ray CT reconstruction was performed on x-ray images obtained from the 150 W and 350 W Ti-6Al-4V samples. Sub-surface voids were characterized by location along the track (y (mm)), void depth (z ($\upmu$m)), and void diameter ($\upmu$m) (Fig. 7). The 150 W sample had an average of 4.1 voids/mm while the 350 W sample had an average of 9.7 voids/mm. For the 150 W sample, the average void diameter was $31.8 \pm 13.4$ $\upmu$m with an average void depth = $178.7 \pm 18.7$ $\upmu$m. For the 350 W sample, the average void diameter was $24.8 \pm 14.7$ $\upmu$m with an average void depth = $465.5 \pm 37.0$ $\upmu$m. Figure 7a is the 3D reconstruction of a section of the 150 W sample (y = 6.00–9.78 mm), where the top and bottom surface of the Ti-6Al-4V sample, melt line, and voids are observable. Figure 7b shows the 2D cross section of the region highlighted in red in Fig. 7a, where five roughly spherical voids are shown as dark circular objects in the lighter Ti-6Al-4V sample. Figure 7b shows the raised melt line as the lighter colored band between the Ti-6Al-4V sample and the background air, seen as the same dark color as the sub-surface voids. The sub-surface voids were analyzed in TXM3DViewer using the 2D viewer and measurement tool. A distinction in both diameter and depth is seen in Fig. 7c between the sub-surface voids produced by the 150 W laser and the 350 W laser. The higher-power laser produced more, smaller, and deeper voids.

## Conclusion

We report on experiments supported by simulations of 100 W, 150 W, and 350 W Ti-6Al-4V LPBF samples to demonstrate a laser-based SAW diagnostic. We show detection of surface defects like spatter and breaks in a melt line as well as sub-surface voids, on single laser melt lines. These LBU results are consistent with independent characterization by optical microscopy (surface features) and X-ray CT (sub-surface voids). This method is well-suited to *in situ* implementation, though there are limits on the size and depth of detectable voids. The minimum detectable void size as a function of depth for this technique is beyond the scope of this report, however, in this study we note that subsurface voids located within 200 $\upmu$m of the sample’s surface were detected and voids located deeper than 400 $\upmu$m were not detected. This depth limitation can be understood by examining the power flux density as a function of depth and acoustic frequency presented here. Additionally, measurements performed on a polished sample that only contained one melt line allowed higher frequency SAW to propagate without attenuating. The single melt line also facilitated interpretation of the scattered displacement field with physical features. The demonstrated all optical SAW technique may find use as an *in situ* diagnostic with a single test melt line made after changes to feed powder or melt laser parameters. Compared to conventional nondestructive evaluation techniques used to study LPBF samples like X-ray CT, the acquisition time for LBU is much quicker, on the scale of minutes, while X-ray CT acquisition with high enough resolution to visualize defects of interest can take several days. Implementation of this diagnostic for in-process monitoring or full post-build inspection requires further development.
